# Improvement and Validation of a Genomic DNA Extraction Method for Human Breastmilk

**DOI:** 10.3390/mps6020034

**Published:** 2023-03-26

**Authors:** Mario Iván Alemán-Duarte, Blanca Rosa Aguilar-Uscanga, Guadalupe García-Robles, Felipe de Jesús Ramírez-Salazar, Israel Benítez-García, Edgar Balcázar-López, Josué Raymundo Solís-Pacheco

**Affiliations:** 1 Laboratorio de Microbiología Industrial, Centro Universitario de Ciencias Exactas e Ingenierías, Universidad de Guadalajara, Blvd, Gral, Marcelino García Barragán 1421, Olímpica, Guadalajara 44430, Mexico; 2 Unidad Académica de Ingeniería en Biotecnología, Universidad Politécnica de Sinaloa (UPSIN), Carretera Municipal Libre Mazatlán Higueras Km 3 Col. Genaro Estrada, Mazatlán 82199, Mexico

**Keywords:** human milk, DNA extraction, DNA quality, microbiota, metagenomics

## Abstract

The human milk microbiota (HMM) of healthy women can vary substantially, as demonstrated by recent advances in DNA sequencing technology. However, the method used to extract genomic DNA (gDNA) from these samples may impact the observed variations and potentially bias the microbiological reconstruction. Therefore, it is important to use a DNA extraction method that is able to effectively isolate gDNA from a diverse range of microorganisms. In this study, we improved and compared a DNA extraction method for gDNA isolation from human milk (HM) samples to commercial and standard protocols. We evaluated the extracted gDNA using spectrophotometric measurements, gel electrophoresis, and PCR amplifications to assess its quantity, quality, and amplifiability. Additionally, we tested the improved method’s ability to isolate amplifiable gDNA from fungi, Gram-positive and Gram-negative bacteria to validate its potential for reconstructing microbiological profiles. The improved DNA extraction method resulted in a higher quality and quantity of the extracted gDNA compared to the commercial and standard protocols and allowed for polymerase chain reaction (PCR) amplification of the V3–V4 regions of the 16S ribosomal gene in all the samples and the ITS-1 region of the fungal 18S ribosomal gene in 95% of the samples. These results suggest that the improved DNA extraction method demonstrates better performance for gDNA extraction from complex samples such as HM.

## 1. Introduction

In metagenomic studies, the objective is to understand the ecological structure of the communities in a given habitat. Therefore, it is crucial to consider the characteristics of the sample, since the high variability of conditions will require methodological considerations to ensure that the sampling, processing, and DNA extraction will accurately reconstruct the identity and abundance of the microorganisms present in the sample, which will reflect the biotic and abiotic interactions of the habitat [1].

Human milk (HM) is the first source of nutrients, bioactive factors, enzymes, and antibodies necessary for a newborn’s survival during the first six months [2]. Previously, this secretion was considered sterile [3]; however, recent research with sequencing tools has reported a complex community of bacteria [4,5,6,7], fungi [8,9,10,11], and archaebacteria [12], either commensalism or mutualistic, whose presence [13] and interaction [14] are related to the healthy establishment of the microbiota in infants [15]. This set of microbial communities is referred to as the human milk microbiota (HMM). The composition of the HMM of healthy women reported in sequencing-based studies varies substantially [14,16,17,18,19,20,21,22,23,24]. The variation may be related to the inherent characteristics of the sample type. However, several reports also showed that the method of collection, storage [25,26,27], and processing [28] might influence the reconstruction of taxonomic profiles [26,28,29].

In addition to the complexity of the matrix (highly rich in proteins and lipids), it is also challenging to consider the low amount of biomass [30] and the high diversity of the HMM (such as Gram-positive, Gram-negative bacteria, fungi, and archaea), whose differences in the composition of the cell wall make some species more difficult to lyse than others [31]. Inadequate lysis of these groups may result in a biased representation of the microbial community in HM samples [25,31,32]. Indeed, these characteristics can interfere with the gDNA isolation process, making HM a relatively complex matrix for high-quality DNA extraction. The methods commonly used to extract gDNA from HM have low efficiency in quantity and quality [25,29,31], directly affecting downstream PCR applications, such as high-quality sequencing, showing the need for optimized microorganism DNA extraction protocols for HM.

To ensure high-quality DNA extraction for downstream sequencing analyses, a set of methods was carefully selected to effectively break down the complex mixture of cell types present in human milk, while minimizing the risk of introducing contaminants or other sources of bias. The first selection was based on the Quick-DNA™ Fecal/Soil Microbe Kit, which has been widely used in previous studies and can isolate gDNA from a broad range of samples. In addition, the guanidinium thiocyanate (GTC) method was chosen for its ability to lyse cells and inactivate RNases to preserve DNA integrity, with both methods utilizing bead-beating to lyse cells. To adapt the CTAB method [33] for use in human milk DNA extraction, the following two modified versions were developed: CTAB-STD and CTAB-2PH. CTAB-STD incorporates CTAB and one round of phenol treatment to reduce potential bias due to the varied composition of cell types, using a combination of mechanical and chemical lysis techniques to rupture the membranes of cells and fat globules. Meanwhile, CTAB-2PH employs an additional round of phenol treatment to further purify the DNA and improve the quality of the reads in sequencing.

This study aimed to improve a method for extracting gDNA from human milk (HM) samples and compare its efficiency to other metagenomic DNA extraction methods. The improved method’s performance was evaluated by comparing the quantity and quality of the isolated DNA, as well as its compatibility with downstream applications such as PCR. Additionally, the ability of the improved method to extract gDNA was tested, from both bacterial and fungal species in HM samples, including culture-dependent microorganisms. The effects of processing on the quality and quantity of gDNA isolated by the improved method were also analyzed.

## 2. Materials and Methods

### 2.1. Human Milk Sampling and Sample Processing

The collection was carried out in the obstetrics department of the civil hospital “Fray Antonio Alcalde’’ (HCFAA) of the University of Guadalajara in Jalisco, Mexico. The HM samples were collected from healthy lactating women. Sampling was performed as follows: donors washed their hands and put on sterile gloves, then the aureola and nipple were disinfected with a 5% chlorhexidine solution, followed by manual discarding of the first drops (0.5–1 mL). After a second cleaning with 5% chlorhexidine solution, 20–50 mL of HM were collected with an electric breast pump, stored in pre-sterilized collection bags (Lansinoh^®^, Alexandria, VA, USA), and labeled. After collection, the samples were transported on ice, and 5 mL aliquots were made. The HM aliquots were centrifuged at 2500× *g* at 4 °C for 20 min, and the fat layer and supernatant were discarded. Subsequently, the cell pellet was washed with TE (10 mM Tris-HCl:1 mM EDTA, pH 8) and concentrated at 20,000× *g* for 20 min. Finally, the new cell pellet was resuspended in 300 μL of TE and stored at −20 °C until further use. This study included 126 mothers who were donors. Mothers provided written consent to participate in the study. The informed consent and confidentiality guidelines were written according to the federal health law on health research [34] and the criteria established by the HCFAA research ethics committee. The donors’ personal information is safeguarded, as stipulated by the federal law for the protection of personal data [35], through official letter No. HCG/CEI-1228/20 with Record 101/20 dated 7 October 2020.

### 2.2. Bacteria and Yeast Growth Conditions

Twenty-three strains were chosen in this study. Ten are Gram-negative bacteria, nine are Gram-positive bacteria, and four are yeasts. Thus, different types of cell wall architecture were represented, emulating the high diversity of microorganisms found in HM samples. The strains and culture conditions used are shown in Table S1. Bacteria were plated on the corresponding culture medium with agar. After confirming Gram staining, isolated colonies were grown aerobically or anaerobically at 37 °C for 16–18 h in 20 mL of culture broth. For yeast strains, cells were plated on a potato dextrose agar (PDA) medium (Neogen, Naucalpan, EM, MX) and grown aerobically at 30 °C for 48 h. Broth cultures were harvested at the end of the exponential growth phase and pelleted by centrifugation at 2500× *g* for 20 min, while yeast cells were collected from the plates. All cell pellets were resuspended in 300 μL TE buffer and stored at −20 °C until further processing.

### 2.3. Cell Counting and Preparation of the Mock Community

Bacterial strains were grown overnight on Luria–Bertani (LB) broth (Invitrogen™, MA, USA) at 37 °C. Cells were collected by centrifugation and then resuspended in TE buffer. All yeast strains were collected from PDA plates and resuspended in TE buffer on ice. The cell density of each strain was determined by spectrophotometry at 600 nm with a GENESYS™ 30 spectrophotometer (Thermo Fisher Scientific, Waltham, MA, USA). We adjusted 1 mL of each strain’s cell density (O.D.) to 0.474 ± 0.015 (~3.198 × 10^8^ cells) by diluting with TE buffer. Aliquots were pelleted by centrifugation at 20,000× *g* at 4 °C for 10 min, after which the supernatant was removed and the pellet resuspended in 300 μL TE buffer. Finally, cell suspensions were processed or stored at −20 °C until further use.

### 2.4. DNA Extraction Methods

Four DNA extraction methods were evaluated using the cell pellets described before, obtained from individual milk samples. Previously, these methods or modifications have been used for gDNA extraction in metagenomic studies of human milk [10,20,36]. Table S2 briefly illustrates the main differences among these extraction methods.

#### 2.4.1. Zymo Method (ZYMO)

The DNA was extracted from the HM cell pellets using the commercially available kit Quick-DNA™ Fecal/Soil Microbe Kit (Zymo Research Corp., Irvine, CA, USA), subsequently called ZYMO, following the manufacturer’s instructions. The milk pellets were added directly to a ZR BashingBead™ Lysis Tube (0.1 & 0.5 mm) and lysed by bead beating. Zymo-Spin™ Technology was then used to isolate the DNA in high concentrations of a chaotropic salt, which was subsequently filtered to remove the humic acids/polyphenols that inhibit PCR. Isolated DNA was then stored at −20 °C until further processing.

#### 2.4.2. Guanidinium Thiocyanate Method (GTC)

This protocol is based on the lysing and nuclease inactivation properties of the chaotropic agent guanidinium thiocyanate (GTC). Briefly, human milk cell pellets were resuspended in 4 mL of lysis buffer (4 M guanidine thiocyanate-Tris 0.1 M, pH 7.5) and 600 mL of 10% N-lauroyl sarcosine). This was grinded with liquid nitrogen and transferred to a microcentrifuge tube, then 500 µL of 5% N-lauroyl sarcosine (0.1 M phosphate buffer, pH 8) was added and incubated at 70 °C for 1 h. After, 750 µL of previously sterilized 0.1 mm diameter silica beads and 15 mg of polyvinylpyrrolidone were added to the tube and vortexed for 10 min. Then, they were centrifuged for 3 min at 20,000× *g* at room temperature (RT). The supernatant was recovered, and the sediment was washed with 500 µL of TENP (50 mM Tris pH 8, 20 mM EDTA pH 8, 100 mM NaCl, 1% polyvinylpyrrolidone), centrifuged for 3 min at 20,000× *g* and the new supernatant was added to the previous one. The washing step was repeated three times. Pooled supernatants were centrifuged briefly to remove particles and divided into two 1.5 mL tubes. Nucleic acids were precipitated by adding 1 volume of isopropanol for 10 min RT and centrifuged for 15 min at 20,000× *g* at 4 °C. Pellets were resuspended and pooled in 450 µL of 100 mM phosphate buffer (pH 8) and 50 µL of 5 M potassium acetate, placed on ice for 90 min and centrifuged at 20,000× *g* for 30 min at 4 °C. The supernatants were transferred to new tubes with 3 µL of RNase (Promega, Madison, WI, USA) per reaction (10 U/μL) and were incubated at 37 °C for 30 min, then 50 µL of 3 M sodium acetate and 1 mL of absolute ethanol were added and incubated for 10 min RT. Then, the mixes were centrifuged at 20,000× *g* for 15 min RT. The DNA pellets were washed with cold 70% ethanol, centrifuged at 20,000× *g* for 5 min RT, dried, and resuspended in 40 µL of TE buffer. Isolated DNA was then stored at −20 °C until further processing.

#### 2.4.3. Cetyltrimethylammonium Bromide (CTAB)-Based Method with Doubled Phenol Extraction (CTAB-2PH)

Genomic DNA was extracted from 5 mL of HM using the CTAB-based method, according to William et al. [33] with some modifications. The HM pellets were thawed and mixed with 100 μL of TE buffer, plus 20 μL of lysozyme (4 U/μL), and incubated for 30 min at 37 °C. Then, 40 μL of 10% sodium dodecyl sulfate (SDS) and 8 μL of *Aspergillus oryzae* proteinase (1 U/μL) were added. The samples were incubated for 3 h at 56 °C. Then, 100 μL of NaCl 5 M and 100 μL of CTAB buffer (100 g CTAB/L, 0.7 M NaCl) were added to the samples and were incubated at 65 °C for 10 min. The samples were extracted twice with 500 μL of phenol:chloroform:isoamyl alcohol (25:24:1) and centrifuged at 20,000× *g* for 10 min RT. The upper phase was transferred to a new tube, and 500 μL chloroform:isoamyl alcohol (24:1) was added and centrifuged at 20,000× *g* for 10 min RT. The aqueous phase was transferred in a new tube, and 0.6 vol of cold isopropanol was added. Samples were incubated overnight (16 h) and centrifuged at 20,000× *g* for 15 min at 4 °C. Pellets were rinsed with cold ethanol (70%) and centrifuged at 20,000× *g* for 5 min RT. The supernatant was discarded, and the pellets were dry RT. Pellets were eluted in 40 µL of DNase-free water. RNase treatment was performed as follows: resuspended DNA was mixed with 4 μL of RNase per reaction (10 U/μL). The mixture was incubated at 37 °C for 1 hr. Subsequently, the enzyme was inactivated at 70 °C for 15 min and cooled with a temperature shock on ice. The precipitation was performed by adding sodium acetate (1/10 volume 3 M) and 2.5 volumes of absolute ethanol to each sample. The samples were mixed gently and kept at −20 °C for 2 h. The samples were centrifuged to 20,000× *g* at 4 °C for 20 min to pellet the DNA. Carefully, the supernatants were removed, and the pellets were washed with cold ethanol (70%). The pellets were centrifuged at 20,000× *g* at 4 °C for 5 min, and the remaining ethanol was removed with a pipet tip. Then, they were dried and resuspended in 40 µL of TE buffer. Isolated DNA was then stored at −20 °C until further processing.

#### 2.4.4. CTAB Standardized for Human Milk Samples (CTAB-STD)

gDNA was extracted from 5 mL of HM from each sample using the CTAB method coupled with phenol-chloroform extraction [33]. An improvement was conducted to achieve maximum DNA extraction with the highest possible purity. HM pellets were thawed and ground into a fine powder using liquid nitrogen. Then, 200 µL of TE at 55 °C was added. Once the powder was dissolved, 40 µL of lysozyme (4 U/µL) was added and mixed by inversion, incubating at 37 °C for 30 min at 150 rpm. After incubation, 30 µL of SDS 10% was added and mixed by inversion carefully (preventing foaming). Then, 16 µL of proteinase from *Aspergillus oryzae* (1 U/µL) was added and mixed by inversion, incubating at 56 °C for 2 h (the samples were shaken approximately every 30 min, or if it was possible, incubated shaking took place at 200 rpm). Later, 100 µL of 5 M NaCl and 100 µL of 10% CTAB pre-warmed to 55 °C were added and mixed by inversion, incubating at 65 °C for 10 min and cooling for 5 min RT, followed by 5 min at 4 °C. After incubation, the tubes were centrifuged at 20,000× *g* for 10 min, and the supernatants were transferred to new tubes. A series of washes were performed with 1 volume of chloroform:isoamyl alcohol (24:1) and centrifuged at 20,000× *g* for 10 min. Then, 4 µL of RNase (10 U/µL) was added, incubating at 37 °C for 1 hr. Subsequently, an extraction with 1 volume of phenol:chloroform:isoamyl alcohol (25:24:1) was performed and centrifuged at 20,000× *g*; again, a wash with chloroform:isoamyl alcohol (24:1) was performed. For DNA precipitation, a volume of isopropanol was used and incubated at −20 °C for two hours. After centrifugation at 20,000× *g* for 15 min, the solution was removed. The generated cell pellet was washed twice with ethanol (70%), allowed to dry, and resuspended in 40 µL of TE buffer. When necessary, the elution was heated at 40 °C for 5 min to improve solubility without physical mixing. Once the sample was tempered, it was properly labeled and stored at −20 °C for later use.

### 2.5. Spectrophotometric Analyses of DNA

In DNA extraction, the concentration and purity of the extracted DNA are important parameters to ensure the accuracy and reliability of downstream analyses. The ratio of absorbance at 260/280 nm and 260/230 nm are commonly used to assess the purity of DNA. The 260/280 ratio reflects the amount of DNA and protein contamination in the sample, with a ratio of ~1.8 indicating pure DNA. The 260/230 ratio reflects the presence of contaminants such as salts and organic compounds, with a ratio of ~2.0 indicating minimal contamination. In this study, the concentration and purity (260/280 and 260/230 ratios) of the extracted DNA were measured with a NanoDrop™ ND-2000 UV spectrophotometer (Thermo Fisher Scientific, Waltham, MA, USA), using 2 µL of each sample.

### 2.6. PCR Amplification

The internal transcribed spacer 1 (ITS1) of nuclear ribosomal DNA from fungi and V3–V4 fragments of 16S rDNA from bacteria were used to verify the amplificability of gDNA. For PCR analysis, each DNA sample was diluted to a working concentration. Amplifications were performed in a MaxyGene II thermal cycler (Axygen^®^, Thermo Fisher Scientific, Waltham, MA, USA). The amplification was performed with the forward primer ITS-1: 5′-TCCGTAGGTGAACCTGCGG-3′ and reverse primer ITS-2: 5′-GCTGCGTTCTTCATCGATGC-3′ for the ITS1 region of the 18S ribosomal gene [37]. The forward primer 341F: 5′-CCTACGGGNGGCWGCAG-3′ and reverse primer 785R: 5′-GGACTACHVGGGTATCTAATCC-’3 were used to amplify the V3–V4 region of the 16S ribosomal gene [38]. The amplification product should be ~200 and ~400 bp, respectively. The oligonucleotides were synthesized and purified by T4OLIGO^®^ (T4OLIGO^®^, Irapuato, GTO, MX). PCR reactions were carried out in a final volume of 25 μL that contained the following: 1 μL of DNA template (0.1–10 ng/μL), 0.5 μL of dNTPs (2.5 mM), 2.5 μL of 10× buffer (25 mM), 0.75 μL MgCl_2_ (25 mM), 1 μL of forward primer and 1 μL of reverse primer, respectively (5 μM), 0.2 μL of Taq DNA polymerase (25 U/mL) (Invitrogen, Waltham, MA, USA), and 18.55 μL of sterile water. PCR thermal cycling conditions were as follows: pre-denaturation at 95 °C for 3 min, followed by 35 cycles of denaturing at 95 °C for 30 s, annealing at 53 °C for 40 s for ITS1 and 55 °C for 40 s for V3–V4 amplicons, extension at 72 °C for 1 min, with a final extension at 72 °C for 5 min. PCR products were stored at −20 °C until further analysis.

### 2.7. Agarose Gel Electrophoresis

The DNA integrity was analyzed by agarose gel electrophoresis using 1% and 2% agarose (TopVision Agarose, Thermo Fisher Scientific, Waltham, MA, USA) for gDNA and PCR amplicons, respectively. Agarose gel was stained with 1 μg/mL of ethidium bromide (EtBr). Electrophoresis was performed using Tris Acetate-EDTA (TAE) buffer and a constant voltage of 100 V for 50 min. The DNA bands were visualized, and images were acquired using a Gel Doc XR+ Imaging system (Bio-Rad Laboratories Inc., Hercules, CA, USA). A DNA sample is considered intact when its profile on agarose gel electrophoresis corresponds to a well-defined “line” of DNA [39]. The level of degradation of a sample is determined by the loss of definition of the predominant band and the accompaniment of a smear along the gel [40]. To assess the integrity of each sample in an objective and standardized way, a measurement scale has been defined for the different DNA profiles observed after the electrophoretic run, as presented in Table S3.

### 2.8. Statistical Analysis

Differences in yield, purity, integrity, and DNA amplification were assessed according to the distribution of the data (Shapiro–Wilk statistic). For parametric data, one-way ANOVA with a Tukey’s honest significant difference (HSD), or a *t*-test, was used. For non-parametric distributions, a Kruskal–Wallis one-way ANOVA with the Mann–Whitney–Wilcox procedure and Bonferroni significance correction post hoc multiple comparisons tests were used. The effect sizes of dependencies were evaluated using the η^2^ coefficient and categorized as a small/medium/large effect size according to Cohen’s conventions [41]. All analyses and graphs used R [42] and Rstudio software [43]. The Microsoft Excel suite was also used to visualize and process data.

## 3. Results

### 3.1. The Improvement of the CTAB-STD Method Increased the Quantity and Quality of the gDNA

**Figure 1:**
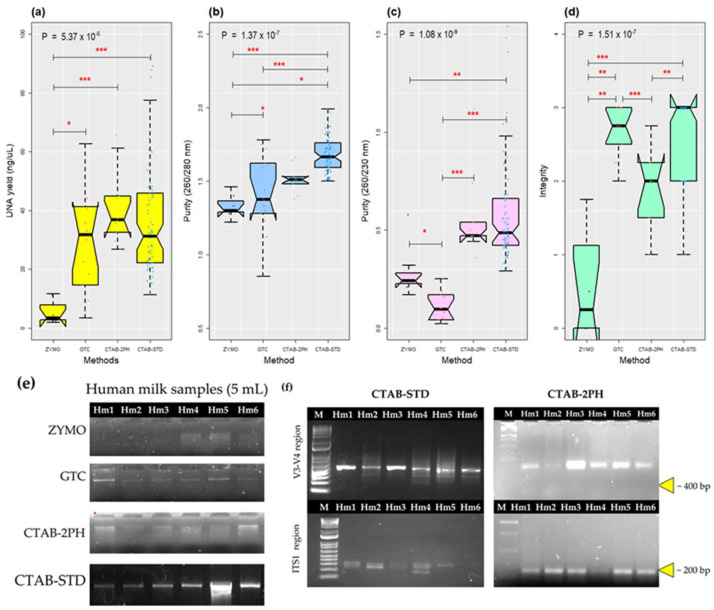
DNA quantity and quality assessment of HM samples. (**a**) Box plot showing the DNA concentration (ng/µL). (**b**) Box plot showing the DNA purity at 260/280 nm. (**c**) Box plot showing the DNA purity at 260/230 nm. (**d**) Box plot showing the DNA integrity. (**e**) Representative results from agarose gel electrophoresis analysis of gDNA from HM samples extracted by four different methods. (**f**) Representative results from agarose gel electrophoresis analysis of PCR amplification (V3–V4 and ITS1 regions from 16S and 18S rRNA, respectively) from Hm samples extracted with CTAB-STD and CTAB-2PH. M represents a gene ruler DNA ladder (1 Kb plus from Invitrogen™, MA, USA). The box signifies the 75% (upper) and 25% (lower) quartiles showing the distribution of 50% of the samples. The line inside the box plot represents the median. The whiskers (top and bottom) represent the maximum and minimum values. Outliers, which are beyond 1.5 times the interquartile range above the maximum value and below the minimum value, are shown with the corresponding color dot. *p*-values for differences between the four methods were calculated using the Kruskal–Wallis statistic. When the difference between methods was significant (*p* < 0.05), all pairwise comparisons were tested for significance using the Mann–Whitney–Wilcox procedure with Bonferroni significance correction. *** represents significance when *p* < 0.001, ** *p* < 0.01 and * *p* < 0.05.

The efficiency of four DNA extraction methods was compared based on the yield, purity, and integrity of the extracted gDNA from the HM samples. The quality of the extracted DNA was verified spectrophotometrically and through agarose gel electrophoresis. The yield of the extracted gDNA was statistically different between the compared methods (*p* < 0.001). The CTAB-2PH (41.42 ± 12.93 ng/µL), CTAB-STD (37.14 ± 22.57 ng/µL), and GTC (29.62 ± 18.07 ng/µL) methods presented similar values. However, the gDNA concentration decreased when the extraction was performed with the commercial package ZYMO (5.39 ± 4.18 ng/µL). Pairwise comparisons indicated a significant decrease in gDNA yield in the ZYMO method compared to all other methods (*P*_ZYMO-GTC_ = 0.0089, *p*_ZYMO-CTAB-2PH_ = 0.0058, *p*_ZYMO-CTAB-STD_ = 9 × 10^−5^), but not between them (Figure 1a). In other words, significant differences were detected in the ZYMO method’s ability to extract gDNA from HM samples, perhaps due to the fact that the matrix is rich in lipids, carbohydrates, and proteins. Furthermore, the ZYMO method used a physical fragmentation that was different from the others by bead beatings.

As assessed by 260/280 nm absorbance ratios, the gDNA purity significantly differed between methods (*p* = 0.001). The CTAB-STD (1.67 ± 0.12) mean ratios were significantly higher than the ratios obtained with CTAB-2PH (1.51 ± 0.09), GTC (1.1 ± 0.26), and ZYMO (1.33 ± 0.08). The lines could be changed as follow The pairwise comparisons revealed significant differences between the methods tested (*p*CTAB-STD-CTAB-2PH = 0.01, *p*CTAB-STD-GTC < 0.001, *p*CTAB-STD-ZYMO < 0.001). Furthermore, the CTAB-2PH method exhibited a statistically significant difference from the ZYMO method (*p*CTAB-STD-ZYMO = 0.048), as illustrated in Figure 1b.

gDNA purity was also assessed using the 260/230 absorbance ratio measure. In addition, significant differences were observed between methods (*p* = 1.08 × 10^−9^). The CTAB-STD (0.58 ± 0.26) mean ratios were significantly higher than those obtained using the ZYMO (0.29 ± 0.14) and GTC (0.11 ± 0.08) methods, as the pairwise comparisons showed (*P*_CTAB-STD-GTC_ =< 0.001, *P*_CTAB-STD-ZYMO_ = 0.00226). The CTAB-2PH (0.56 ± 0.22) method significantly differed from the ZYMO and GTC methods (*P*_CTAB-STD-ZYMO_ = 0.00022, *P*_CTAB-STD-ZYMO_ = 0.01818). The ZYMO method differed from the GTC method (*P*_ZYMO–GTC_ = 0.01690). There was no significant difference in DNA purity between CTAB-STD and CTAB-2PH (Figure 1c). According to the purity results (260/280 and 260/230 nm ratio), the CTAB-based methods, which were free from guanidine contamination, were associated with the highest (most desirable) values.

The integrity of extracted gDNA was assessed by agarose gel electrophoresis (Figure 1d). In order to ensure the accuracy and consistency of the quantitative evaluation of DNA integrity, the evaluation was calculated by three different analysts. This was to minimize the potential for bias or errors that could arise from individual observations or interpretations. Each analyst independently evaluated the DNA integrity using a scale from 0 to 3, as described in the methods section (Table S3). This allowed for multiple evaluations of the same sample, which were then compared and averaged to arrive at a final score. Gel electrophoresis revealed that the CTAB-STD, CTAB-2PH, and GTC extracted DNA with more integrity compared to the ZYMO method (Figure 1d). The introduction of modifications in the CTAB-STD method attained statistical significance in performance compared with the other methods (*p* < 0.001), showing the highest value, as the pairwise comparisons tests showed (*P*_GTC-ZYMO_ =< 0.001, *P*_CTAB-2PH-ZYMO_ = 0.0298, *P*_CTAB-STD-ZYMO_ =< 0.001, *P*_GTC-CTAB-2PH_ = 0.0092, *P*_CTAB-2PH–CTAB-STD_ = 0.0012). The lowest amount and highest degradation of DNA were observed when the ZYMO method was used. Moreover, four out of seven samples (0.57 prevalence) extracted by the ZYMO method yielded no detectable gDNA with agarose gel electrophoresis (Figure 1e). No statistically significant differences were detected in the integrity of the samples when processed with the GTC method (even visually, they presented more defined bands than the CTAB methods and no smears). However, the prevalence was lower compared to the CTAB-STD method, showing samples with no bands. Regrettably, all of the samples processed using the GTC method failed the amplificability tests, including the V3–V4 (16S rRNA), fungal ITS1 (18S rRNA), and restriction with multiple enzymes (Figure S1). Consequently, we also decided to discontinue the use of the GTC method. Table 1 summarizes the DNA concentration, purity (at O.D. 260/280 and 260/230), and integrity obtained for all the HM samples using the four extraction methods. Although the sample’s characteristics might influence purity and the amount of extracted DNA in this experiment, the samples were randomly subjected to different extraction methods to minimize the matrix effect. Therefore, the observed variations can be attributed to differences in the DNA extraction methods.

A total of 84 out of 105 DNA samples from human milk (HM) were extracted using both the CTAB-2PH and CTAB-STD methods. These samples were then analyzed by end-point PCR that targeted the V3–V4 and ITS1 regions of gene markers. Interestingly, the CTAB-STD method amplified the V3–V4 region in all the samples, while ITS1 amplified 95% of the samples. In CTAB-2PH, all samples were amplified for the V3–V4 fragment and ITS1 amplified 80% of all the samples (Figure 1f). Considering the demonstrated advantages in terms of quantity, quality, and amplifiability for the CTAB-STD method, we decided to proceed with its validation. These results suggest that the CTAB-STD method reported here improves the quantity and quality of gDNA extracted from HM, showing a better performance than the commercial and standard methods previously applied to similar samples. The amount and quality of DNA extracted from a sample are relevant because they directly influence its amplificability and, consequently, the taxonomic resolution of metagenomes.

### 3.2. CTAB-STD Method Can Extract a High Quantity and Quality of gDNA from Fungi, Gram-Positive, and Gram-Negative Bacteria

**Figure 2:**
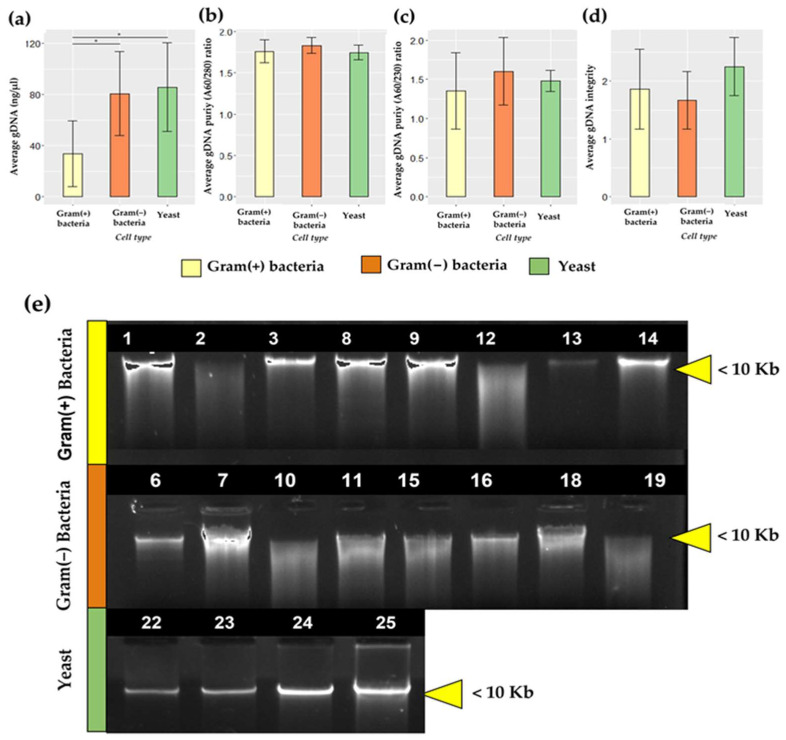
Quantity and quality of DNA from microorganisms isolated using the CTAB-STD method. (**a**) Bar plots showing the DNA concentration (ng/µL). (**b**) Bar plots showing the DNA purity at 260/280 nm. (**c**) Bar plots showing the DNA purity at 260/230 nm. (**d**) Bar plots showing the DNA integrity of the three cell types assessed. (**e**) Representative results from gel electrophoresis analysis of gDNA from microorganisms isolated using the CTAB-STD method. The bar signifies the mean. The line inside the bars represents the standard deviation. *p*-values for differences between the different cell types were calculated using an ANOVA statistic. When the difference between regions was significant (*p* < 0.05), all pairwise comparisons were tested for significance using the Tukey–Kramer multiple comparisons test (HSD). * Represents significance was *p* < 0.05. Yellow represents values for Gram(+) bacteria, orange represents values for Gram(−) bacteria, and green represents values for yeast.

The HMM is constituted of fungi, archaeobacteria, Gram-positive and Gram-negative bacteria [44]; therefore, an efficient DNA extraction method must be able to extract the total amount of DNA present in a complex sample from this wide range of organisms, whose cell wall composition differs considerably. The CTAB-STD method optimized for the HM samples effectively extracted DNA from isolated cultures of yeast, Gram-positive, and Gram-negative bacteria (Figure 2e). Table 2 summarizes the DNA concentration, purity (at O.D. 260/280 and 260/230), and integrity obtained for all microorganism samples. The DNA yield resulted in a statistical difference between cell types (*p* < 0.001), suggesting an influence of the cell wall composition of the microorganisms on DNA extraction.

The DNA yield was lower in Gram-positive bacteria (33.74 ± 25.83 ng/µL) than in Gram-negative bacteria (80.68 ± 32.89 ng/µL) and yeasts (85.86 ± 34.84 ng/µL). The mean yield DNA values were statistically significant when compared between Gram-positive and Gram-negative bacteria (*p* = 0.0205, η^2^ = 0.61, large effect size) and Gram-positive and yeast (*p* = 0.0394, η^2^ = 0.39, medium effect size), but not between Gram-negative and yeast. There was no significant change in DNA purity for both 260/280 (*p* = 0.528) and 260/230 nm ratios (*p* = 0.4454). Figure 2b,c show that the average values of all the measured samples fell within the range of 1.5 to 1.9 for the 260/280 nm ratio and 0.6 to 2.0 for the 260/230 nm ratio, respectively. Although the statistical analysis did not show significant differences in DNA integrity due to cell type (Figure 2d), a trend of lower DNA integrity in Gram-negative bacteria compared to Gram-positive bacteria and yeast was observed in the agarose gel shown in Figure 2e. This observation will be further discussed in the following section. The amplificability test did not show significant differences.

### 3.3. Effects of Mechanical Fragmentation on DNA Extraction from Different Cell Types

In the section above, the results suggested that the CTAB-STD method effectively extracted more gDNA from Gram-negative bacteria than Gram-positive or yeast. A multifactorial design with repeated measures was used to test whether there was a trend to extract more gDNA from organisms with different cell wall compositions and whether sample processing could affect the quantity and quality of gDNA. The factors used were as follows: (i) organism type, with three levels, Gram-positive bacteria (Gram(+) bacteria) and Gram-negative bacteria (Gram(−) bacteria) and yeast as a focal variable, (ii) physical fragmentation, with two levels, macerated with liquid nitrogen (N2(+)) and not macerated (N2(−)), and (iii) freezing process, with two levels, those corresponding to a storage time of five months and five freeze/thaw (FT) cycles and those corresponding to fresh samples with only one FT cycle (fresh), with the latter two factors as moderating variables. Samples were standardized to an average O.D. of 0.47 ± 0.01 at 600 nm. The expected concentration was calculated based on each bacterial genome’s size, the base pair’s weight, and each bacterium’s colony-forming unit (CFU).

**Figure 3:**
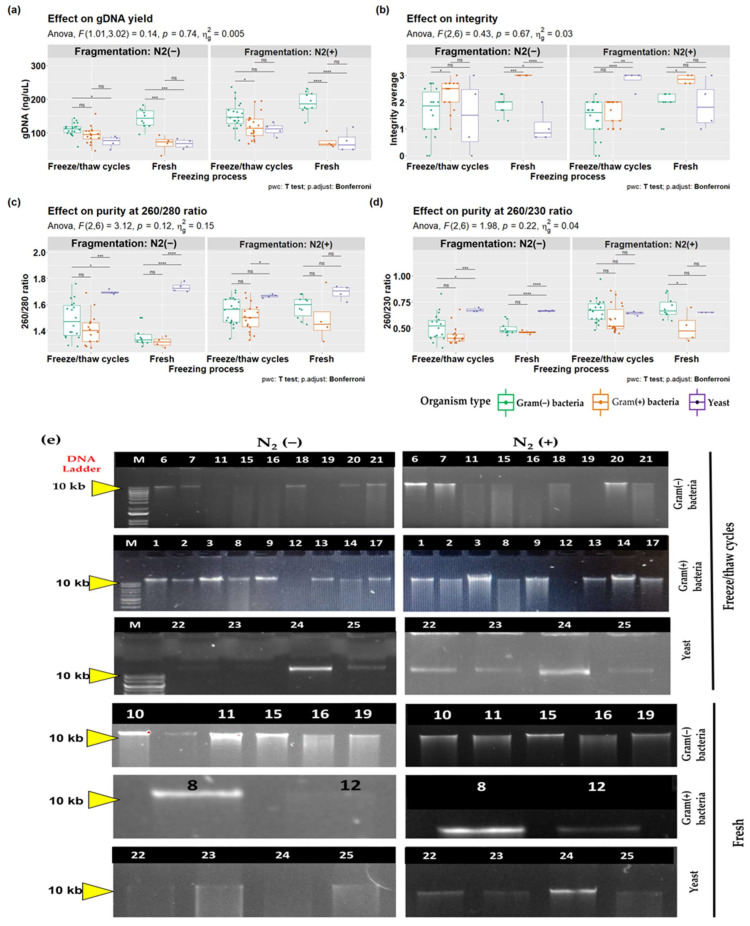
Effect of processing on DNA quantity and quality from isolated microorganism samples. (**a**) Box plots showing the DNA concentration (ng/µL). (**b**) Box plots showing the DNA purity at 260/280 nm. (**c**) Box plots showing the DNA purity at 260/230 nm. (**d**) Box plots showing the DNA integrity. All boxplots show the yeast, Gram(+) and Gram(−) bacteria grouped by processing and fragmentation. (**e**) Representative results from gel electrophoresis analysis of gDNA from microorganisms isolated using the CTAB-STD method. N2(+) represents maceration with liquid nitrogen, and N2(−) represents extraction without maceration. M represents a gene ruler DNA ladder (1 Kb plus from Invitrogen™, MA, USA). The box signifies the 75% (upper) and 25% (lower) quartiles showing the distribution of 50% of the samples. The line inside the box plot represents the median. The whiskers (top and bottom) represent the maximum and minimum values. Outliers, which are beyond 1.5 times the interquartile range above the maximum value and below the minimum value, are shown with the corresponding color dot. *p*-values for differences between the treatments were calculated using a three-way ANOVA statistic. When the difference between regions was significant (*p* < 0.05), all pairwise comparisons were tested for significance using a *t*-test procedure with Bonferroni significance correction. **** for *p* < 0.0001, *** for *p* < 0.001, ** *p* < 0.01, and * *p* < 0.05.

The results suggested that there is no statistically significant effect for the three-way interaction for DNA yield (*p* = 0.74). However, there was a statistically significant two-way interaction between the freezing process and organism type, only for N2(+) (*p* = 0.03), but not for N2(–) (0.02). The simple effects of organism type and fragmentation also resulted in significance (*p* = 0.02 and *p* < 0.001, respectively) for N2(+) and N2(−). The results indicate that the effect of treatment on DNA yield using the CTAB-STD method varies depending on the type of organism and the level of physical fragmentation applied. When the simple effect was calculated for the focus variable, a statistically significant increase in DNA yield (153 ± 38.9 ng/µL) was observed for Gram(−) bacteria macerated with N2(+) and subjected to multiple FT cycles (*p* < 0.001). In contrast, the effect was not observed for Gram(+) bacteria (67.7 ± 25.9 ng/µL) or yeast (67.6 ± 14.3 ng/µL), without maceration or fresh samples (*p* = 0.118). These results highlight the relevance of the physical fragmentation process for the DNA yield of HM samples, which is an improvement of the method presented in this study. Pairwise comparisons showed a tendency to decrease significance within FT cycles (*P*_N2(−)_ = 0.012 and *P*_N2(+)_ = 0.036), suggesting a reduction in DNA yield bias due to organism differences. These results indicate that when multiple FT cycles and liquid nitrogen are used during sample processing, the significant difference in DNA yield decreases, which would allow us to avoid bias in the reconstruction of microbiological profiles due to the difference in organism type (Figure 3a).

Purity at the 260/280 nm ratio showed a range between 1.32 ± 0.03 and 1.73 ± 0.03 in all conditions, without a statistically significant effect for the three-way interaction (*p* = 0.12). There was a statistically significant two-way interaction between the freezing process and fragmentation (*p* = 0.002) in only Gram(−) bacteria (*p* < 0.001), suggesting that the variation observed is influenced by the type of organism used in the DNA extraction. In the same way, when simple effects were analyzed, Gram(−) and Gram (+) showed a significant decrease when they were fresh and not macerated with liquid nitrogen (1.36 ± 0.07 and 1.32 ± 0.03) compared with yeast (1.73 ± 0.036), which showed the highest purity. With the application of nitrogen liquid maceration, the pairwise comparisons indicated a decrease in significant differences attributed to cell type (Figure 3b). Thus, it is inferred that the decrease in purity might be due to the lack of effective cell fragmentation.

The purity at the 260/230 nm ratio did not show any significant differences for the three-way interaction (*p* = *0*.22), but there were significant differences for the two-way interaction between fragmentation and organism type (*p* = 0.025). Once again, nitrogen liquid maceration led to a reduction in significant differences related to cell type. Simple effects analysis revealed that fragmentation at the N2(−) level was a significant variable (*p* < 0.001) for Gram(−) bacteria in both FT cycles (*p* < 0.001) and fresh samples (*p* < 0.001), as well as for yeast in fresh samples (*p* = 0.014). On the other hand, fragmentation at the N2(+) level was only significant for Gram(+) bacteria (Figure 3c).

No significant difference was observed for DNA integrity in the three-way interaction (*p* = 0.67). The two-way interaction between fragmentation and FT cycles showed a significant difference only for Gram(+) bacteria (*p* = 0.008), increasing integrity in all cases compared to the Gram(−) bacteria. In the fresh condition, an increase in integrity was observed, although it was not statistically significant in the comparisons. The pairwise comparisons indicated a significant difference between the Gram(+) and Gram(−) bacteria when macerated, compared to when they were not macerated (*p* = 0.007). Using liquid nitrogen and multiple FT cycles resulted in a loss of integrity. The situation was exacerbated if the organisms were Gram(−) bacteria (*p* < 0.001). Integrity results are crucial for this method since losing the integrity of the genetic material during extraction may contribute to the loss of species that are naturally underrepresented in complex samples (Figure 3d,e).

## 4. Discussion

The present study evaluated four DNA extraction methods from HM samples. Sample quantity and quality were based on spectrophotometer measurements and agarose gel electrophoresis scores. According to the DNA yields obtained with the different methods, a significant reduction was observed when using ZYMO (*p* =< 0.001) compared to the GTC- and CTAB-based methods (Figure 1a). It is important to emphasize that DNA yield in the HM extractions is challenging to compare since it depends not only on the efficiency of the extraction method, but also on the quantity and characteristics of the sample itself. In the CTAB-STD method, a mean DNA yield of 37.14 ± 22.57 ng/µL was obtained, which, compared with previously reported results, is considerably lower [25,45,46]. The previously mentioned publications used either a single human milk sample or a pool. Although this allows for a decrease in the variability caused by the sample and measures the influence of the extraction method on the DNA yield, the method’s effectiveness in authentic sampling is masked. Therefore, in improving the CTAB-STD method, it was necessary to evaluate the efficacy of the extraction method in genuine random sampling so that the results reported here present greater robustness.

For gDNA to be considered pure, an absorbance ratio of 260/280 nm equal to or greater than 1.80 is required. The 260/280 ratio in the CTAB-STD method had an average of 1.68 ± 0.12 (ranging from 1.5 to 1.9), showing a significant difference compared to GTC, CTAB-2PH, and ZYMO (*p* < 0.001). This result reveals that the modifications improved DNA extraction with acceptable purity ratios for further use in downstream applications. Previous efforts have been made to optimize the purity of DNA extractions in their 260/280 ratio, showing similar or lower results to those reported in this study [25,28,47]. A 260/230 nm ratio of less than 2.0 indicates contamination with organic compounds [48]. In this study, all the methods tested obtained values below 2, ranging from 0.29 to 1.54. However, within the observed values, the CTAB-STD method obtained the best performance with an average ratio of 0.58 ± 0.26 and a significant difference compared to the GTC and ZYMO methods (*p* < 0.05). According to previously published literature [25,49], where the performance of different commercial DNA extraction methods has been evaluated, similar 260/230 rate results have been obtained to those reported in this study. These low 260/230 rate levels may be due to the high levels of fat present in HM, which can negatively influence the efficacy of DNA isolation buffers [27,28]. Moreover, various components of the matrix, such as polysaccharides and polyphenols, or chemicals from extraction, might considerably influence the 260/230 rates [50].

Agarose gel electrophoresis is a commonly used criterion for assessing DNA integrity. The results of the CTAB-STD method suggest a significant increase (*p* < 0.001) in integrity compared to the other methods evaluated here (Figure 1d). However, there are few studies where the integrity of the evaluated samples is reported. In this regard, Gaur et al. showed gels where a characteristic smear is observed along the entire electrophoretic run [49], a particularity observed in the CTAB-2PH method. It was minimized during the improvement process for the HM samples. Thus, the CTAB-STD method showed superior performance in the integrity of the DNA extracted from the HM samples (Figure 1e). These results suggest that the purity, integrity, and amount of DNA are higher in HM samples when using the CTAB-STD method. However, it is essential to note that the 260/230 rate needs to be raised to avoid errors in subsequent analyses and improve the resolution level of HM research.

The amplifiability test revealed the ability of the CTAB-STD method to amplify the V3–V4 fragment in all the HM samples and the fungal ITS1 fragment in 95% of the samples. This stands in contrast to that presented by Moossaavi et al., where only 21.4% of the amplified samples were reported [10]. When reviewing the extraction method, it was observed that they used a commercial package [20] from ZYMO (Quick-DNA^™^ Fungal/Bacterial Kit), whose basis is guanidine thiocyanate in its lysis buffer and fragmentation with beads. In this study, a similar commercial package (Quick-DNA^™^ Fecal/Soil Microbe Kit) was used, but it demonstrated the worst performance across all the methods [10]. Another study by Boix-Amorós et al. obtained a higher prevalence of positive fungal samples, reporting a range between 35% and 80% [9], which is even lower than the one reported here for the CTAB-STD method. The extraction method included an InviMag^®^ Stool DNA Kit (Stratec Molecular, BE, DE), specifying a mechanical and chemical lysis treatment. Similarly, the CTAB-STD method uses mechanical and chemical lysis with proteinases to increase cell wall fragmentation. However, the CTAB-STD method improves the performance given the complexity of the sample, low biomass, and high diversity of organisms with different cell structures. These results are relevant because they suggest that the previously reported fungal metagenomes could be biased due to the DNA extraction method used, a situation that can be solved using the CTAB-STD proposed in this study. Nevertheless, further studies should be carried out.

Different organisms that mimic HM diversity were selected to evaluate the CTAB-STD method to extract gDNA from microorganisms with different cell wall compositions, such as yeasts, Gram(+), and Gram(−) bacteria. In addition, isolated cultures were evaluated for the quality and quantity of the DNA extracted. Purity at the 260/280 rate reaches the optimal range (1.83 ± 0.09) without a significant difference between the type of organisms (*p* = 0.20). On the other hand, purity at the 260/230 rate (1.47 ± 0.35) does not reach the optimal value, but is considerably higher than that obtained in the HM samples (0.58 ± 0.26). This result suggests that the complexity of the HM samples substantially contributes to the low value observed in the 260/230 rate. Of all the literature consulted, no other publication uses isolated cultures to evaluate the amplificability of a DNA extraction method. Previous studies have used synthetic communities to assess the efficiency of different methods, but have not reported the effect on the purity and integrity of the extractions [28,31]. DNA yield achieved a significant reduction (*p* =< 0.05) in Gram(+) (33.74 ± 25.83 ng/µL) compared with Gram(−) (80.68 ± 32.89 ng/µL) bacteria and yeasts (85.86 ± 34.84 ng/µL), suggesting that the difference in cell wall composition may have affected the DNA yields. In a previous study that evaluated the efficacy of different DNA extraction methods to reconstruct a microbiological profile, an underrepresentation of Gram(+) bacteria was observed [28,51], particularly bacteria of the genus *Lactobacillus* and *Streptococcus,* which have cell walls that are difficult to break down [31]. The results reported in a previous section suggest a significant decrease in the DNA extraction of Gram(+) bacteria (Figure 2a), which could explain the Gram(+) underrepresentation reported.

A three-factor experiment was designed to obtain a broader picture of what might influence this observed difference, including yeast, Gram(+) and Gram(−) bacteria, liquid nitrogen as a cell fragmentation mechanism, and multiple FT cycles. All cultures were standardized to an optical density of 0.47 ± 0.13 at 600 nm, and two different analysts duplicated the extractions. The results of the three-way ANOVA suggest that none of the interactions for any of the response variables (DNA yield, purity, and integrity) is statically significant. However, in all the response variables, an interaction between fragmentation and FT cycles was observed (*p* < 0.001), particularly in the result obtained in Gram(+) bacteria, which tend to be decreased in most combinations compared to Gram(−) bacteria. Previously reported results precisely suggested an underrepresentation of Gram(+) bacteria when reconstructing microbiological profiles of HM samples [28,29,30,52].

Interestingly, when comparing the results of fresh samples without maceration to samples macerated with liquid nitrogen and exposed to multiple FT cycles, the significant difference in DNA yield between yeast and Gram(+) with Gram(−) bacteria reaches its minimum value, suggesting that both maceration and FT cycles contribute to fragmenting the cells and increasing DNA concentrations. A study by Lyons et al. that evaluated the effect of storage, extraction methods, and temperature concluded that the most effective method for accurately reconstructing the biological community they considered was temporary storage at −80 °C; furthermore, the extraction methods included physical fragmentation [26]. Similarly, the results reported by Xin et al. showed that storage at −20 °C and three FT cycles were the best freezing condition and could markedly enhance DNA extraction efficiency, while also preserving the species diversity of meconium microbiota [46]. Another study by Männistö et al. on artic bacterial communities showed that FT cycles do not drastically change the microbiological profiles of the samples [45].

There were no significant differences in the purity at 260/280 between the Gram(+) and Gram(−) bacteria under any condition. In comparison to yeast, however, the purity at 260/280 was significantly higher. Additionally, the samples that were subjected to multiple FT cycles showed an even greater increase in purity when compared to fresh samples. This suggests that fragmentation plays a role in the removal of protein residues from the sample, which is further amplified in the combined effect. In addition, purity at a rate of 260/230 shows a significant improvement (*p* < 0.001) in comparison between the type of organism when using any fragmentation method, suggesting that the modifications made to the CTAB-STD method substantially contribute to improving the quality of DNA extraction. In contrast to previous observations, integrity appears to decrease in all cases when using either fragmentation method, showing the worst performance in combining these two factors, with Gram(−) bacteria being the most affected (1.36 ± 0.687). On the other hand, the worst condition for yeast was found to be fresh without maceration, which suggests that the composition of the cell wall could have an influence on the results.

Taken together, these results explain some previously reported findings [26,46,53] in which an overrepresentation of Gram(−) bacteria was observed, as they are more susceptible to fragmentation than their Gram(+) counterparts, suggesting that there may be a bias in the microbiological reconstructions of the HM samples. The representative gel in Figure 3e shows how integrity is more affected in Gram(−) bacteria than in Gram(+) bacteria or yeast. These results highlight the need for precautions when processing and storing complex samples where a mixture of organisms may be present.

## 5. Conclusions

It is often challenging to extract microbial DNA from milk samples using commercial DNA extraction kits due to the low microbial biomass and the presence of organic components. The CTAB-STD method improved the yield, purity, and integrity of the extracted DNA. DNA extraction methods for complex samples and metagenomic purposes should be able to extract total DNA from a wide range of species [52]. This study demonstrates that the CTAB-STD method can effectively extract DNA from yeasts, Gram(−), and Gram(+) bacteria. In addition, the proposed method was able to amplify all samples for the V3–V4 fragment and 95% of the HM samples for the fungal ITS1 fragment, indicating its potential for use in metagenomic applications to identify fungi as part of the HMM.

Furthermore, optimizing freeze–thaw cycles can increase the total DNA extracted from the sample, minimizing fragmentation and the impact of variations in cell wall composition. Proper storage, processing, and extraction of HM samples can contribute to the quantity, purity, and integrity of gDNA samples for metagenomic purposes. In summary, this work presents a protocol to improve the extraction of bacterial and fungal DNA from HM samples, which may provide insights into their ecological dynamics and vulnerability to changes due to the internal and external conditions of the mothers.

## Figures and Tables

**Table 1:** Summary of the quantity and quality of human milk DNA extraction by the DNA extraction method.

DNA Extraction Method	ZYMO		GTC		CTAB-2PH		CTAB-STD		^†^ *p*-Value
	*Mean ± SD*	*Median*	*Mean ± SD*	*Median*	*Mean ± SD*	*Median*	*Mean ± SD*	*Median*	
DNA (ng/µL)	5.39 ± 4.18	3.4	29.62 ± 18.07	36.85 _f_	41.42 ± 12.93	31.65 _e_	37.14 ± 22.57	31.19 _c_	5.37 × 10^−5^
Purity (260/280 nm)	1.33 ± 0.08	1.3	1.1 ± 0.26	1.38	1.51 ± 0.09	1.51 _e_	1.68 ± 0.12	1.67 _a,b,c_	1.38 × 10^−7^
Purity (260/230 nm)	0.29 ± 0.14 _f_	0.24	0.11 ± 0.08	0.1	0.56 ± 0.22	0.47 _d_	0.58 ± 0.26	0.48 _a,b_	1.09 × 10^−9^
Integrity	0.61 ± 0.8	0.25	2.68 ± 0.36	2.75	1.9 ± 0.58	2 _d_	2.64 ± 0.54	3 _a,c,e,f_	1.51 × 10^−7^

Values are means ± standard deviation (SD). **^†^** *p*-values for differences between the four methods were calculated using the Kruskal–Wallis statistic. When the difference between regions was significant (*p* < 0.05), all pairwise comparisons were tested for significance using the Mann–Whitney–Wilcox procedure with Bonferroni significance correction. _a_ The value for the variable with the CTAB-STD method significantly differs from that of ZYMO (*p* < 0.001). **_b_** The value for the variable with the CTAB-STD method significantly differs from that of GTC (*p* < 0.001). **_c_** The value for the variable with the CTAB-STD method significantly differs from that of CTAB-2PH (*p* < 0.001). **_d_** The value of the variable with the CTAB-2PH method significantly differs from that of GTC (*p* < 0.01). **_e_** The value of the variable with the CTAB-2PH method significantly differs from that of ZYMO (*p* < 0.001). **_f_** The value of the variable with the GTC method significantly differs from that of ZYMO (*p* < 0.001). ZYMO: Quick-DNA Fecal/Soil Microbe Kit from ZYMO research^®^; GTC: guanidinium thiocyanate method; CTAB-2PH: cyltrimethylammonium bromide double phenol step; CTAB-STD: cyltrimethylammonium bromide standardized for human milk; ng: nanograms; µL: microliters; nm: nanometers.

**Table 2:** Summary of the quantity and quality of DNA extraction by cell type using the CTAB-STD method.

Cell Type	DNA (ng/µL)		Purity (260/280 nm)		Purity (260/230 nm)		Integrity	
	*Mean ± SD*	*Median*	*Mean ± SD*	*Median*	*Mean ± SD*	*Median*	*Mean ± SD*	*Median*
Gram(+) bacteria	33.74 ± 25.83 _a,b_	28.15	1.76 ± 0.14	1.83	1.35 ± 0.49	1.49	1.86 ± 0.69	2
Gram(−) bacteria	80.68 ± 32.89 _c_	60.34	1.83 ± 0.09	1.86	1.6 ± 0.43	1.68	1.67 ± 0.5	2
Yeast	85.86 ± 34.84	97.22	1.75 ± 0.09	1.75	1.48 ± 0.14	1.48	2.25 ± 0.5	2
*p*-value	0.0133 ^†^		0.528 ^¥^		0.4454 ^¥^		0.2663 ^¥^	

Values are means ± standard deviation (SD). **^†^** *p*-values for differences between the four response variables were calculated using ANOVA statistics. When the difference between regions was significant (*p* < 0.001), all pairwise comparisons were tested for significance using the Tukey–Kramer honest significant difference (HSD) test. ^¥^ *p*-values for differences between the four response variables were calculated using the Kruskal–Wallis statistic. When the difference between regions was significant (*p* < 0.05), all pairwise comparisons were tested for significance using the Mann–Whitney–Wilcox procedure with Bonferroni significance correction. **_a_** The value for the variable in Gram(+) bacteria significantly differs from that of Gram(−) bacteria (*p* < 0.01). **_b_** The value for the variable in Gram(+) bacteria significantly differs from that of yeast (*p* < 0.01). **_c_** The value of the variable in Gram(−) bacteria significantly differs from that of yeast (*p* < 0.01). ng: nanograms; µL: microliters; nm: nanometers; ns: non-significant.

## Data Availability

Not applicable.

## Supplementary Materials

The following supporting information can be downloaded at: https://www.mdpi.com/article/10.3390/mps6020034/s1; Supporting information_1 contains: Table S1: Strains and cultivation conditions used in this study; Table S2: Summary of DNA extraction methods used in this study; Table S3: DNA quality based on electrophoresis of genomic DNA, Supporting information_2 contains a step by step protocol of the improved CTAB method of DNA extraction for human milk samples. Supporting information_3: Supplementary Figure S1. References [33,54,55,56,57,58,59,60,61,62,63,64,65,66,67,68,69,70,71,72,73,74,75,76] are cited in the Supplementary Materials.
