# Supplementary material for: Improvement and Validation of a Genomic DNA Extraction Method for Human Breastmilk
Source: Methods Protoc. 2023 Mar 26;6(2):34. doi: 10.3390/mps6020034 (PMC10144544; doi:10.3390/mps6020034)
Supplement: Supplementary file 1 [file mps-06-00034-s001.zip › Supporting information_1_mps-2162081.pdf]

Supporting information

Supplementary Table S1

Table S1 Strains and cultivation conditions used in this study

ID	Strain	Gram-stain	Genome size (Mbp)	plasmid (kbp)	Atmosphere [a]	Medium[b]	Reference
Bacteria strains							
1	*Lactobacillus plantarum*	+	3.20	0	Anaerobic	MRS:Agar and broth	[54]
2	*Lactobacillus fermentum*	+	1.90	32.6	Anaerobic	MRS:Agar and broth	[55]
3	*Staphylococcus aureus*	+	2.80	0	Aerobic	LB: Agar and broth	[56]
6	*Bordetella bronchiseptica*	-	5.20	0	Aerobic	LB: Agar and broth	[57]
7	*Citrobacter freundii*	-	4.96	0	Aerobic	LB: Agar and broth	[58]
8	*Bacillus cereus*	+	5.50	208	Aerobic	LB: Agar and broth	[59]
9	*Staphylococcus hominis*	+	2.34	0	Aerobic	LB: Agar and broth	[60]
10	*Salmonella typhimurium*	-	5.10	265	Aerobic	LB: Agar and broth	[61]
11	*Providencia stuartii*	-	4.42	0	Aerobic	LB: Agar and broth	[62]
12	*Staphylococcus epidermis*	+	2.45	15.14	Aerobic	LB: Agar and broth	[63]
13	*Micrococcus sp. KBS0714*	+	2.42	0	Aerobic	LB: Agar and broth	[64]
14	*Staphylococcus aureus* ATCC 6538	+	2.82	28	Aerobic	LB: Agar and broth	[65]

15	*Enterobacter cloacae*	-	4.78	208.74	Aerobic	LB: Agar and broth	[66]
16	*Escherichia coli ATCC 8739*	-	5.40	0	Aerobic	LB: Agar and broth	[67]
17	*Staphylococcus haemolyticus*	+	2.63	41.6	Aerobic	LB: Agar and broth	[68]
18	*Morganella morganii*	-	3.86	0	Aerobic	LB: Agar and broth	[69]
19	*Proteus mirabilis*	-	4.01	102	Aerobic	LB: Agar and broth	[70]
20	*Klebsiella pneumoniae*	-	5.59	180	Aerobic	LB: Agar and broth	[71]
21	*Serratia marcescens RSC-14*	-	5.12	0	Aerobic	LB: Agar and broth	[72]
Yeast strains							
22	*Naganishia sp (HMI)*	NA	Unknown	NA	Aerobic	PDA	In preparation
23	*Candida albicans*	NA	14.69	NA	Aerobic	PDA	[73]
24	*Candida tropicalis*	NA	15.3	NA	Aerobic	PDA	[74]
25	*Rhodotorula*	NA	18.6	NA	Aerobic	PDA	[75]

[a]Anaerobic strains were cultivated in GasPak anaerobic chamber (Becton Dickinson, Franklin Lakes, NJ) with Pack-Anaero sachet (MGC Inc., NY, NY, USA).

[b]Medium: LB, Luria-Bertani (LB agar: 10 g NaCl, 10 g tryptone, 5 g yeast extract per liter, pH 7.2. Add 1.5% agar. Sterilize by autoclaving.); MRS, De Man, Rogosa and Sharpe (Oxoid, Basingstoke, HA, UK); PDA, potato dextrose agar.

HMI, Human milk isolated; NA, not apply;

Supplementary Table S2

Table S2 Summary of DNA extraction methods used in this study

Method name	Code	Basis and format	Starting material	Cell lysis	Removal of contaminants	DNA precipitation	Elution buffer	Reference
Quick-DNA™ Fecal/Soil Microbe Kit	ZYMO	Solid-based; Silica based and ZymoSpin™ Technology	Cell pellet from 5 mL of mature human milk	ZR BashingBead ™ in a Lysis solution	Silica matrix	ZymoSpin™ Technology	20 µL DNA Elution Buffer	(Zymo Research, Tustin, CA, USA)
Guanidinium thiocyanate	GTC	Solution-based; Guanidinium thiocyanate	Cell pellet from 5 mL of mature human milk	Bead-Beating method with a lysis buffer (4M guanidine thiocyanate, Tris 0.1M, pH 7.5 y 600 µL of N-Lauroylsarcosine)	TENP (Tris 50 Mm, pH 8, EDTA 20 Mm, pH 8; NaCl 100 Mm, 1% polyvinylpyrrolidone)	1/10th volume of sodium acetate from 3M pH5.2 and 2.5-3 vols ice cold 100% Ethanol	40 µL sterile deionized water	Modify from [76]
Cetyltrimethylamonium bromide double phenol step	CTAB-2PH	Solution-based; selective precipitation of DNA with CTAB (10%).	Cell pellet from 5 mL of mature human milk	Chemical and enzymatic lysis with SDS 10% and lysozyme (100 mg/mL)	Washing with organic solvents, two strong (phenol:chloroform:isoamyl alcohol), and one weak (chloroform:isoamyl alcohol)	Ice cold 0.6 volume of isopropanol	40 µL sterile deionized water	Modified from [33]

Cetyltrimethylamonium bromide standardized for human milk	CTAB-STD	Solution-based; selective precipitation of DNA with CTAB (10%).	Cell pellet from 5 mL of mature human milk	Mechanical lysis using liquid nitrogen (N_2 +). Chemical lysis with SDS 10% coupled with lysozyme (100 mg/mL)	Washing with organic solvents, one strong (phenol:chloroform:isoamyl alcohol), and two weak (chloroform:isoamyl alcohol)	Ice cold Isopropanol (v/v)	40 μL TE buffer (10 mM Tris hydrochloride, 1 mM EDTA, pH 8.0)	This study

mg, miligrams; Ml, mililiters; mM, miliMolar, v/v, volumen/volumen; μL, microliters;

Supplementary Table S3

Table S3 DNA Quality based on electrophoresis of genomic DNA

Electrophoretic characteristics	Electrophoretic Integrity	Indicator description	Assigned score
DNA quality must be assessed in 1% agarose gel	High integrity	Well defined line at the top of the gel (Discrete band)	3
	Adequate integrity	Simultaneous presence of a partially discrete band at the top with slight smearing	2
	Partially degraded	No discrete band and the presence of a concentrated smear at the top and along the entire electrophoretic run.	1
	Fully degraded	No band, smear is concentrated at the bottom or not observed	0

Adapted from "Programa de control de calidad de ácidos nucleicos. Carlos III National DNA Bank (University of Salamanca). www.bancoadn.org"
