# Supplementary material for: Improvement and Validation of a Genomic DNA Extraction Method for Human Breastmilk
Source: Methods Protoc. 2023 Mar 26;6(2):34. doi: 10.3390/mps6020034 (PMC10144544; doi:10.3390/mps6020034)
Supplement: Supplementary file 1 [file mps-06-00034-s001.zip › Supporting information_2_mps2162081.pdf]

Protocol for DNA extraction from human milk using CTAB-STD method

1 Introduction:

The present protocol delineates a systematic methodology for the extraction of DNA from human milk utilizing the CTAB method. Human milk comprises a plethora of living cells such as viruses, fungi, bacteria Gram-positive and Gram-negative, macrophages, neutrophils, lymphocytes, epithelial cells, and fat globules encased by a hydrophilic membrane consisting of cholesterol, phospholipids, glycoproteins, and enzymes. To effectively isolate gDNA from these diverse cell types, the protocol employs a combination of mechanical and chemical lysis techniques to rupture the membranes of cells and fat globules. This approach is further optimized by incorporating CTAB and phenol, which helps in mitigating any potential bias resulting from the varied composition of cell types.

Objective

Isolate high molecular weight genomic DNA with little or no fragmentation and low contamination of residues that may affect downstream analysis or sequencing.

Table S1

Reactants concentrations
Lysozyme (100 mg/mL).
SDS 10%
Proteinase *A. oryzae* (10 mg/mL);
RNAse A (10 mg/mL)
Phenol:Chloroform:Isoamyl alcohol (25:24:1)
Chloroform:Isoamyl alcohol: (24:1)
CTAB (10% w/v)*#
NaCl 5M
EDTA 0.5M
Tris-HCl 1M
TE buffer (1:10)
Isopropanol
Ethanol 70%

*use freshly made solution; heat to 55-60°c before adding CTAB
#CTAB = Cetyl Trimethyl Ammonium Bromide (Sigma m-7635).

2 Procedure

2.1 Sample preparation for extraction (homogenization)

1. Take 5 mL of fresh cold milk and centrifuge at 4,500 rpm at 4°C.
2. Separate the phases into clean sterile tubes (lipids, serum, and pellet).
3. Label and store the fractions at -20°C.
4. Add 300 μL of TE 1:10 at room temperature (RT) to the tube with the pellet and lipid remains.

5. Centrifuge at 13,000 rpm for 10 min at 4°C.
6. Remove supernatant.
7. Elute in 100 µL of TE1:10 RT.
8. Macerate in liquid nitrogen, adding the elution liquid with a pipette.

2.2 Tissue or cell disruption

1. Take the powder from maceration with liquid nitrogen and resuspend in 200 µL of TE1:10 at 55°C (note: it is important to generate a thermal shock).
2. Add 40 µL of Lysozyme (100 mg/mL). Mix by inversion intensely for two min. Incubate for 30 min at 37°C.
3. Add 30 µL of 10% SDS and mix by inversion.
4. Add 16 µL of proteinase *A. oryzae* (10 mg/mL); mix slowly by inversion (to avoid bubbles).
5. Incubate at 56°C for 2 hours.

2.3 CTAB extraction

9. Add 100 µL of 5M NaCl and mix by shaking vigorously.
10. Add 100 µL of CTAB (10%) and mix (preheated at 55°C).
11. Incubate in a water bath at 65°C for 10 min (can also be done in an incubator).
12. Incubate for 5 min at RT and 5 min on ice.

2.4 Phenol chloroform treatment

13. Add 500 µL of Chloroform:Isoamyl alcohol: (24:1). Mix by inversion.
14. Centrifuge at 20,000 xg for 10 min at RT
15. Recover the supernatant and transfer to a new tube.
16. Add 4 µL of RNase A.
17. Mix by inversion and incubate at 37°C for 1 hour (**caution:** you should mix every 15 min or, if not, incubate with agitation at 150 rpm).
18. Add 500 µL of Phenol:Chloroform:Isoamyl alcohol (25:24:1), mix by shaking vigorously.
19. Centrifuge at 20,000 xg for 10 min at RT
20. Recover supernatant and transfer to a clean tube.
21. Add 500 µL Phenol:Chloroform:Isoamyl alcohol (25:24:1), mix vigorously.
22. Centrifuge at 20,000 xg for 10 min at RT.
23. Recover supernatant and transfer to a clean tube.
24. Note: if the liquid phase contains impurities or appears turbid, repeat centrifugation at 20,000 xg for 15 min at RT and again remove the transparent phase directly with a pipette, avoiding any visible impurities. Repeat as many times as necessary before proceeding with the washes.
25. Add 500 µL Chloroform:Isoamyl alcohol (24:1) cold, mix vigorously.

26. Centrifuge at 20,000 xg for 10 min at RT.
27. Recover supernatant and transfer to a clean tube.

2.5 DNA precipitation and elution

28. 29. Add 350-400 µL of cold isopropanol (-20°C) to each tube and mix gently by inversion.
29. Let the samples rest for 120 min at -20°C (it is not recommended to exceed the freezing time, we found that the longer the resting time, the lower the purity).
30. Centrifuge at 20,000 xg for 15 min at 4°C.
31. Remove the isopropanol and add 700 µL of cold 70% ethanol (-20°C).
32. Centrifuge at 20,000 xg for 5 min at 4°C.
33. Remove the ethanol and centrifuge a pulse to remove the remaining ethanol with a micropipette.
34. Leave the samples to dry on the rack for 10 min to evaporate the ethanol (be careful not to overdry, the time is approximate. It is advisable to keep an eye on the ethanol evaporation).
35. Add 40 µL of TE1:10 and incubate at 55°C for 10 min with gentle agitation (avoid pipetting to prevent fragmentation of high molecular weight DNA). If the extraction was effective, you should not need this step. If you observe that the pellet does not dissolve correctly when adding TE1:10, it is advisable to perform this step.
36. Label properly and store at -20°C (for immediate use) or at -80°C (for storage for months).

3 Conclusion:

This protocol provides a simple and effective method for DNA extraction from human milk using CTAB/SDS method. The extracted DNA can be used for various downstream applications, such as PCR, qPCR, sequencing, and genotyping.
