# Supplementary material for: Improvement and Validation of a Genomic DNA Extraction Method for Human Breastmilk
Source: Methods Protoc. 2023 Mar 26;6(2):34. doi: 10.3390/mps6020034 (PMC10144544; doi:10.3390/mps6020034)
Supplement: Supplementary file 1 [file mps-06-00034-s001.zip › Supporting information_3.pdf]

Supplementary figure S1

Improvement and validation of a genomic DNA extraction method for human breastmilk

Methods and Protcols, MDPI

Test to amplify V3-V4 fragment from GTC extractions

A1

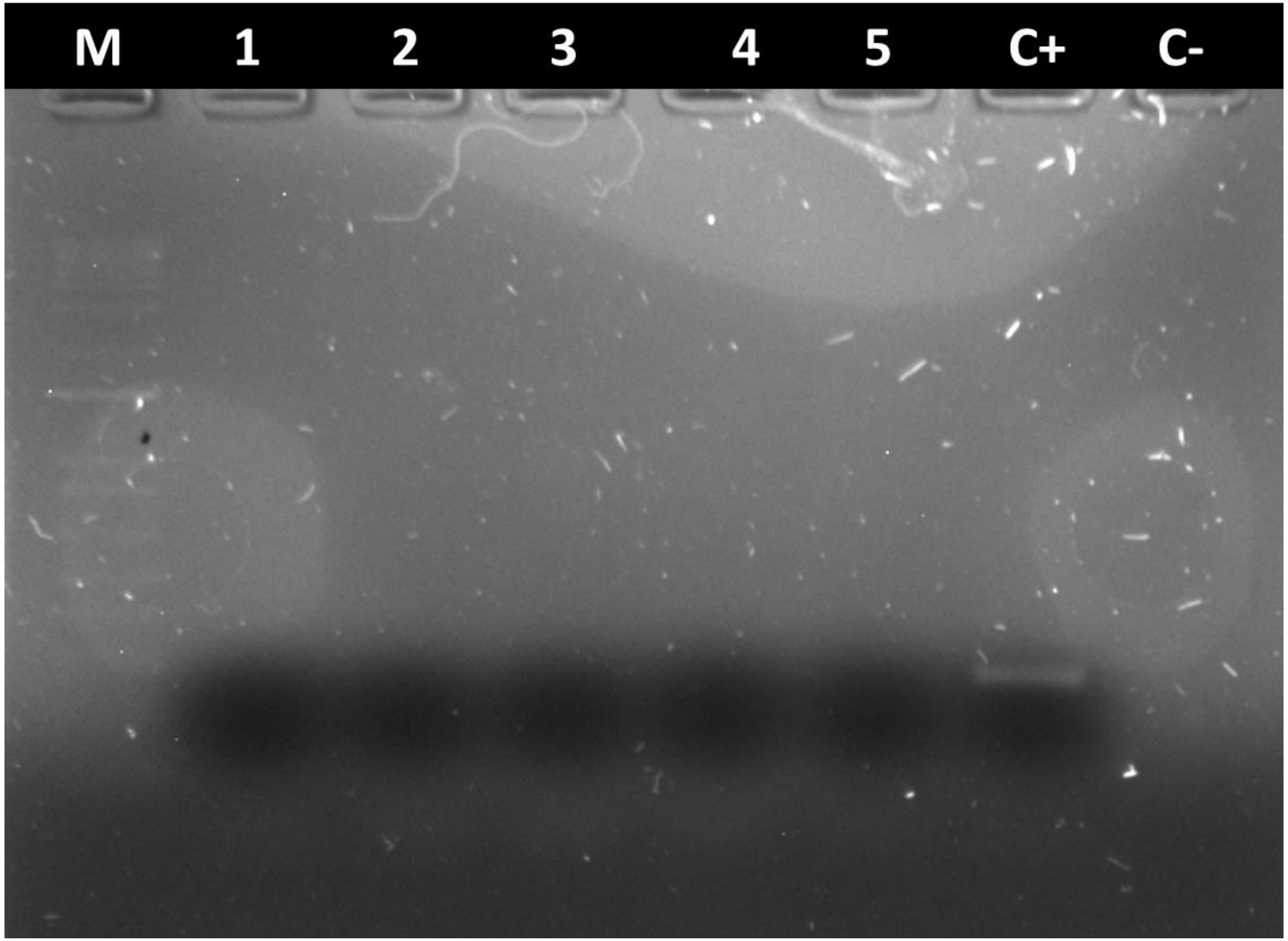

Five samples were randomly selected from extraction #1 of human milk samples

Agarose gel electrophoresis:
80 Vots, 46 min
4% agarose gel
Expected DNA-amplicón ~400 pb
Forward: 341F: 5’-CCTACGGGNGGCWGCAG-3’
Reverse: 785R: 5’-GGACTACHVGGGTATCTAATCC-’3
M = 1kb plus (Invitrogen)
1-5 = Random samples from first extraction
C+ = DNA extracted from bacteria culture with GTC method
C- = Amplification without gDNA

Test to amplify V3-V4 fragment from GTC extractions

A2

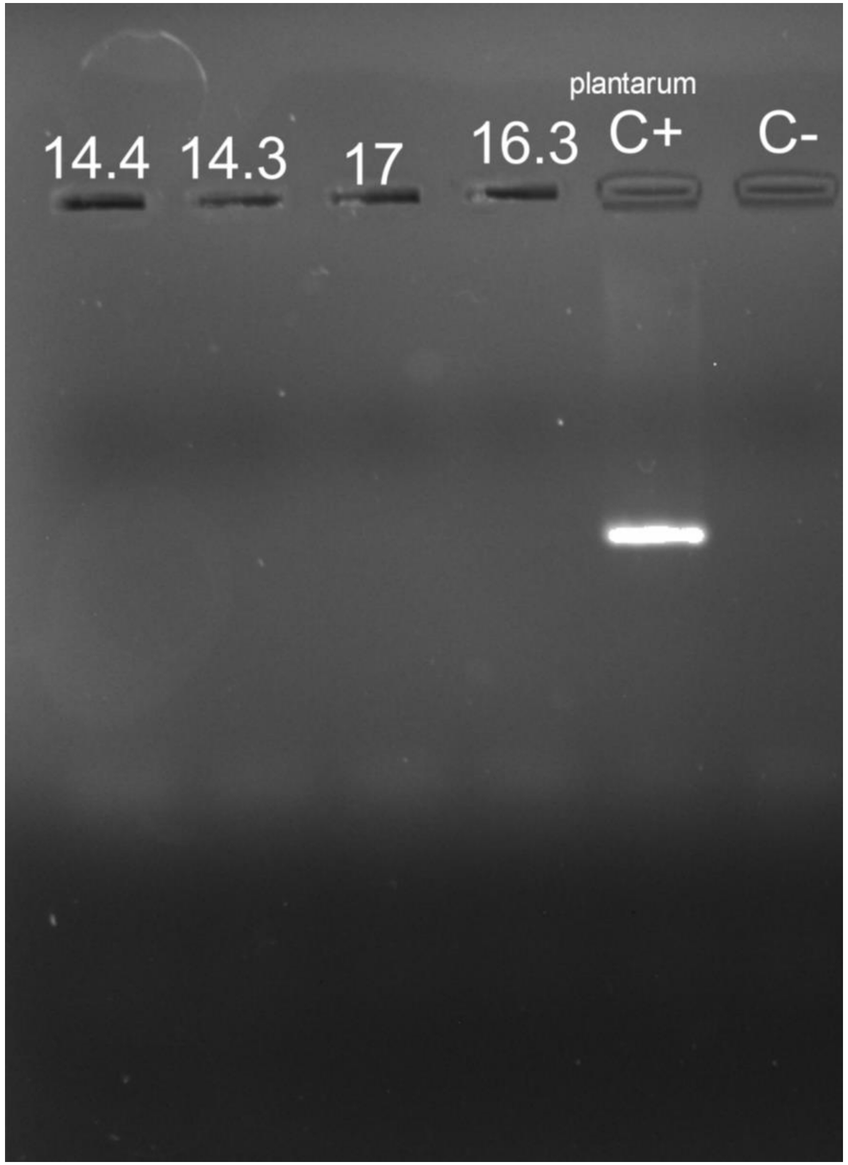

Samples randomly selected from all extraction attempts
14.4 Sample #14 GTC method, attempt 4
14.3 Sample # 14 GTC method, attempt 3
17 Sample # 17 GTC method, attempt 1
16.3 Sample # 16 GTC method, attempt 3
C+ Fresh culture from *Lactobacillus plantarum* GTC method
C- Amplification without gDNA

Agarose gel electrophoresis:
80 Vots, 46 min
4% agarose gel
Expected DNA-amplicón ~400 pb
Forward: 341F: 5′-CCTACGGGNGGCWGCAG-3′
Reverse: 785R: 5′-GGACTACHVGGGTATCTAATCC-′3

Test to amplify ITS1 fragment from GTC extractions

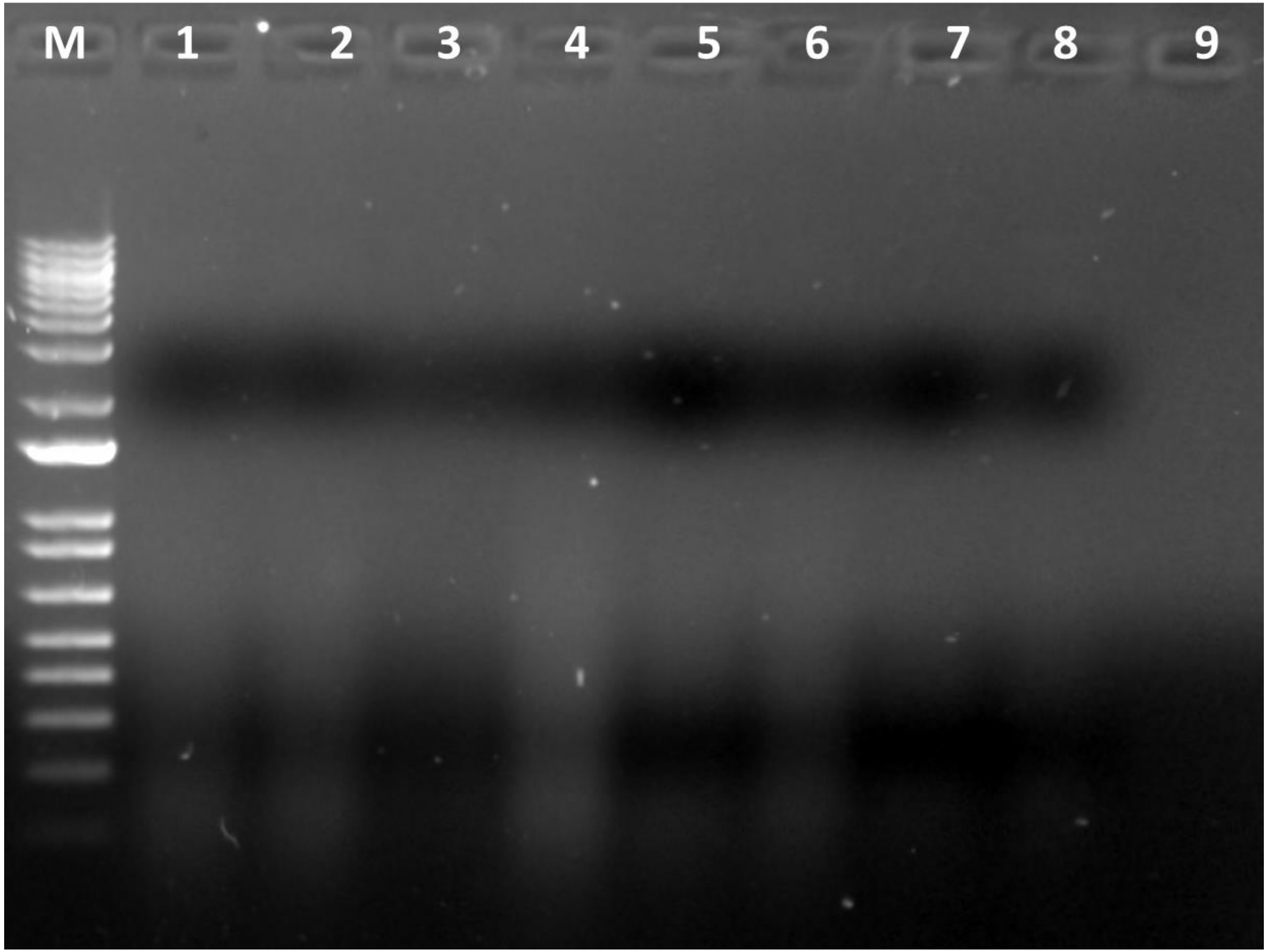

Samples randomly selected
from the first extraction

Agarose gel electrophoresis:
80 Volts, 46 min
4% agarose gel
Expected DNA-amplicón ~200 pb
Forward: ITS-1: 5′-TCCGTAGGTGAACCTGCGG-3′
Reverse: ITS-2: 5′-GCTGCGTTCTTCATCGATGC-3′
1-9 Samples were randomly selected to show ITS-1 amplicons

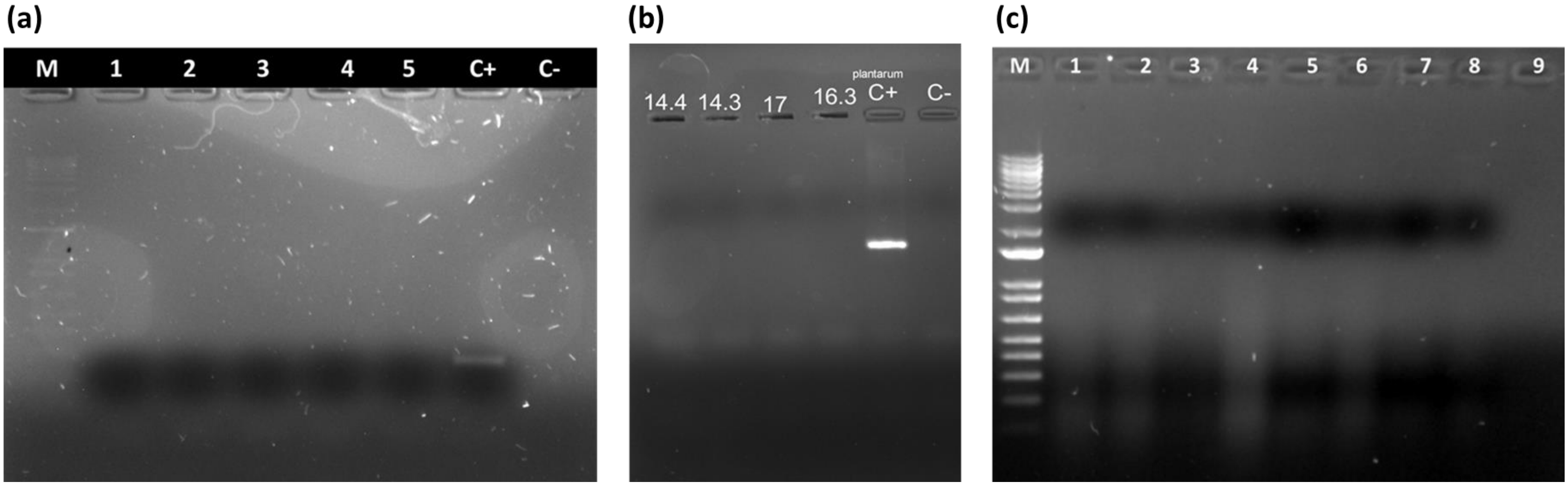
(a)
M 1 2 3 4 5 C+ C-
(b)
14.4 14.3 17 16.3 plantarum C+ C-
(c)
M 1 2 3 4 5 6 7 8 9

Supplementary Figure S1. The figure shows representative results from gel electrophoresis analysis of PCR-amplicons obtained from human milk DNA samples using the GTC method. Panel (**a**) displays the results of the V3-V4 DNA-amplicon test, where wells 1 to 5 contain samples randomly selected from the first extraction. The positive control (C+) shows amplification of the V3-V4 fragment of *Lactobacillus plantarum* using the GTC method, while the negative control (C-) shows amplification without gDNA. Panel (**b**), presents results from the V3-V4 DNA-amplicon test with samples marked as 14.4, 14.3, 17, and 16.3, which were randomly selected from different gDNA extraction attempts to test the V3-V4 amplification. The positive control (C+) shows amplification of the V3-V4 fragment of *Lactobacillus plantarum* using the GTC method, while the negative control (C-) shows amplification without gDNA. Panel (**c**) shows the results of the ITS-1 DNA-amplicon test, where wells 1 to 9 contain samples randomly selected from the first extraction. Finally, a gene ruler DNA ladder (1Kb plus from Invitrogen™, MA, USA) is included in all panels and denoted by M.

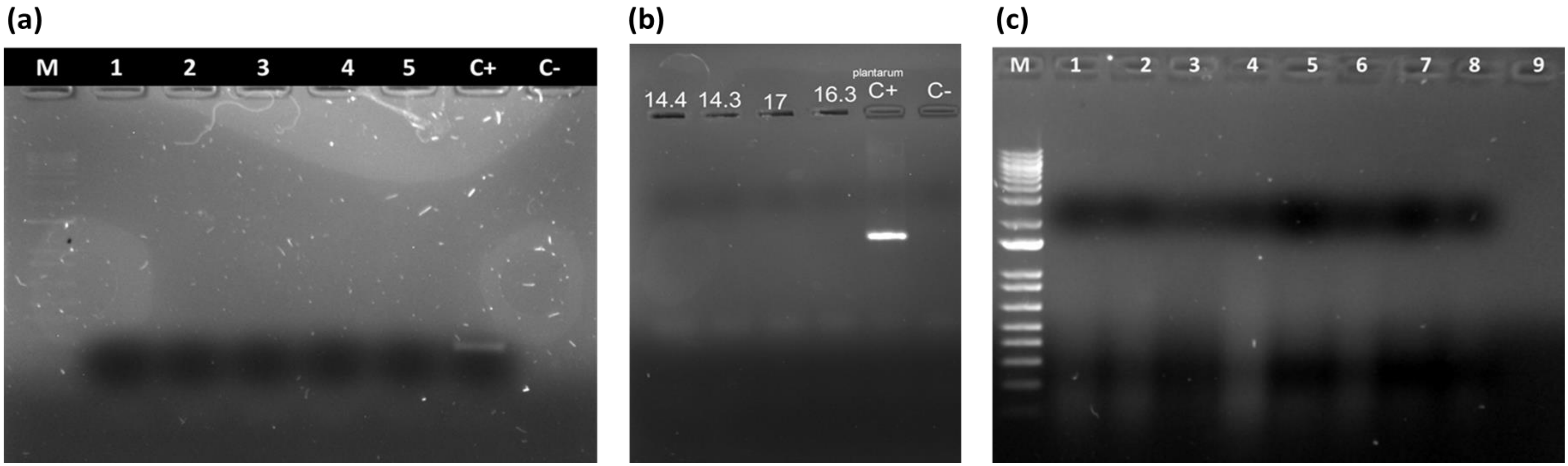

Figure S1. The figure shows representative results from agarose gel electrophoresis analysis of PCR-amplicons obtained from human milk DNA samples using the GTC method. Panel (**a**) displays the results of the V3-V4 DNA-amplicon test, where wells 1 to 5 contain samples randomly selected from the first extraction. The positive control (C+) shows amplification of the V3-V4 fragment of *Lactobacillus plantarum* using the GTC method, while the negative control (C-) shows amplification without gDNA. Panel (**b**), presents results from the V3-V4 DNA-amplicon test with samples marked as 14.4, 14.3, 17, and 16.3, which were randomly selected from different gDNA extraction attempts to test the V3-V4 amplification. The positive control (C+) shows amplification of the V3-V4 fragment of *Lactobacillus plantarum* using the GTC method, while the negative control (C-) shows amplification without gDNA. Panel (**c**) shows the results of the ITS-1 DNA-amplicon test, where wells 1 to 9 contain samples randomly selected from the first extraction. Finally, a gene ruler DNA ladder (1Kb plus from Invitrogen™, MA, USA) is included in all panels and denoted by M.
